# Content validity and clinical meaningfulness of the HFMSE in spinal muscular atrophy

**DOI:** 10.1186/s12883-017-0790-9

**Published:** 2017-02-23

**Authors:** Maria C. Pera, Giorgia Coratti, Nicola Forcina, Elena S. Mazzone, Mariacristina Scoto, Jacqueline Montes, Amy Pasternak, Anna Mayhew, Sonia Messina, Maria Sframeli, Marion Main, Robert Muni Lofra, Tina Duong, Danielle Ramsey, Sally Dunaway, Rachel Salazar, Lavinia Fanelli, Matthew Civitello, Roberto de Sanctis, Laura Antonaci, Leonardo Lapenta, Simona Lucibello, Marika Pane, John Day, Basil T. Darras, Darryl C. De Vivo, Francesco Muntoni, Richard Finkel, Eugenio Mercuri

**Affiliations:** 1 Pediatric Neurology, Catholic University, Largo Gemelli 8, 00168 Rome, Italy; 2 Dubowitz Neuromuscular Centre, UCL Institute of Child Health & Great Ormond Street Hospital, London, UK; 3 Department of Neurology, Columbia University Medical Center, New York, NY USA; 4 Department of Neurology, Boston Children’s Hospital, Harvard Medical School, Boston, MA USA; 5 John Walton Muscular Dystrophy Research Centre, Institute of Genetic Medicine, Newcastle University, Newcastle, UK; 6 Department of Clinical and Experimental Medicine and Nemo Sud Clinical Center, University of Messina, Messina, Italy; 7 Stanford University, Palo Alto, CA USA; 8 Nemours Children’s Hospital, University of Central Florida College of Medicine, Orlando, USA

**Keywords:** Spinal muscular atrophy, Quality of life, Carers, Clinical trials

## Abstract

**Background:**

Reports on the clinical meaningfulness of outcome measures in spinal muscular atrophy (SMA) are rare. In this two-part study, our aim was to explore patients’ and caregivers’ views on the clinical relevance of the Hammersmith Functional Motor Scale Expanded- (HFMSE).

**Methods:**

First, we used focus groups including SMA patients and caregivers to explore their views on the clinical relevance of the individual activities included in the HFMSE. Then we asked caregivers to comment on the clinical relevance of possible changes of HFMSE scores over time. As functional data of individual patients were available, some of the questions were tailored according to their functional level on the HFMSE.

**Results:**

Part 1: Sixty-three individuals participated in the focus groups. This included 30 caregivers, 25 patients and 8 professionals who facilitated the discussion.

The caregivers provided a comparison to activities of daily living for each of the HFMSE items.

Part 2: One hundred and forty-nine caregivers agreed to complete the questionnaire: in response to a general question, 72% of the caregivers would consider taking part in a clinical trial if the treatment was expected to slow down deterioration, 88% if it would stop deterioration and 97% if the treatment was expected to produce an improvement.

Caregivers were informed of the first three items that their child could not achieve on the HFMSE. In response 75% indicated a willingness to take part in a clinical trial if they could achieve at least one of these abilities, 89% if they could achieve two, and 100% if they could achieve more than 2.

**Conclusions:**

Our findings support the use of the HFMSE as a key outcome measure in SMA clinical trials because the individual items and the detected changes have clear content validity and clinical meaningfulness for patients and their caregivers.

**Electronic supplementary material:**

The online version of this article (doi:10.1186/s12883-017-0790-9) contains supplementary material, which is available to authorized users.

## Background

Several efforts have been made recently to identify disease specific outcome measures for spinal muscular atrophy (SMA) patients. The Hammersmith Functional Motor Scale Expanded (HFMSE), a motor function scale specifically designed for SMA, is widely used in patients. [1–3] The activities included in the original Hammersmith scale and in the expanded version were chosen by clinicians because of their functional relevance after careful observation and evaluation of many SMA patients [1–3].

The potential for therapeutic benefit from interventions in SMA has highlighted the need to obtain reliable documented evidence of patient input to support the clinical meaningfulness of the measures used in natural history studies and in clinical trials [4–7].

The activities included in the HFMSE have been found to be extremely useful in clinical practice as an assessment and rehabilitation tool, and in natural history studies and clinical trials to establish disease progression [8–15]. However, no systematic study has been performed to determine if the individual activities included in the scale are also relevant for patients and their caregivers.

This approach, to include the patient perspective, has been strongly encouraged by the United States Food and Drug Administration (FDA) [16]. This regulatory agency has indeed suggested that patient reported scales should be used to determine the relevance of the observed functional changes [17].

One of the challenges is that SMA is clinically very heterogeneous; and, even when restricted to the type 2 and 3 phenotypes whose functional domains are covered by the HFMSE, the clinical severity still ranges from non-ambulant sitting patients with only a few points on the scale to ambulant patients who may be able to complete nearly all of the 33 items on the scale [8, 9].

Another significant challenge is the variability of SMA types 2 and 3 disease progression, as reported by recent natural history studies, and the various factors, such as age or functional level, that influence different trajectories [10].

Because of these and other challenges, it is may be difficult to determine a clinically meaningful change for patients at different ages and at different functional levels. Moreover, it is not clear if similar quantitative improvements in the scale, two points for example, have the same clinical meaning regardless of where the patients score on the HFMSE scale.

This paper describes a two part study reporting: (1) patients’ and caregivers’ view on the clinical relevance of the HFMSE, and (2) the possible changes of HFMSE scores over time. More specifically, in the first part we aimed to explore caregivers’ and patients’ views on the clinical meaningfulness of each individual HFMSE item, asking them to describe the implications between the activity being explored in the individual items and the consequences as it relates to activities of daily living. In the second part we collected caregivers’ views on the relevance of possible changes on the HFMSE scale. The novelty of our approach was that, rather than just asking general open questions, we tailored them according to each participant’s specific functional level based on their HFMSE score.

## Methods

### Part 1: Content validity of the HFMSE

The first part was based on patients’ and caregivers’ focus groups as we explored content validity of individual HFMSE items. This qualitative study was conducted in Italy between June and October 2015 as part of a collaborative project with the two main Italian SMA advocacy groups (Famiglie SMA and Asamsi). The study was approved by the Ethical Committees of all the participating centers (Catholic University, Rome; University of Messina, Messina; UCL Institute of Child Health & Great Ormond Street Hospital, London; Columbia University Medical Center, New York; Harvard Medical School, Boston; Newcastle University, Newcastle; Stanford University; University of Central Florida College of Medicine, Orlando). Four focus groups were completed during the annual conventions of both advocacy groups. Three of the 4 focus groups included caregivers and one also included patients. The participants volunteered to be part of these activities and signed a dedicated consent form. No compensation was provided for their participation.

Patients and caregivers were given a form describing the items of the HFMSE, in lay language, illustrating the activities included in the scale with some pictures. They were then asked to comment on the relevance of the individual items, whether each activity assessed in the items could be related to activities of daily living, and if and why this was relevant to them.

Each focus group was run by a psychologist and a member of our team (clinician or Physical therapist) who transcribed the responses immediately before moving to the next item.

The results of the various groups were analyzed by assigning a code to each response and by identifying consistencies across the various groups tabulating the frequency of individual responses in the various subgroups.

### Part 2: Clinical meaningfulness of HFMSE changes

The aim for this part was to establish the view of the caregivers on the clinical relevance of HFMSE changes in relation to their children’s functional level. This could only be performed in patients who had a recent clinical functional assessment. This study was part of an international effort.

From September 2015 to April 2016, we administered a questionnaire or conducted semi-structured telephone interviews with caregivers of type 2 and 3 SMA patients.

All consecutive patients attending our clinics, who routinely underwent functional assessments, were included. Telephone interviews were only conducted if patients had been seen within the previous 3 months and if the results of their functional assessments were available.

All centers shared the same training and had already performed inter-observer reliability for the HFMSE [10]. Study participants did not receive any form of compensation. Caregivers were first asked to provide general information regarding the patients’ disease course over the last year and their expectations for the near future.

An innovative aspect of this questionnaire was the introduction of specific questions that were related to the subjects’ motor performance as assessed by the standardized HFMSE functional scale. In the scale the items follow a hierarchical order with increasing difficulty, from top to bottom, built on the frequency distribution of findings observed in a large cohort of SMA patients The score on the scale provides a clear indication of the patient’s functional level, and the subsequent activities represent activities likely to be achieved.

The advantage of this approach is that caregivers are asked questions about activities that are realistically close to their child’s possible achievements, rather than generic questions on other activities, such as walking or running for non-ambulant type 2 SMA patients, that clearly would be highly desirable but difficult or impossible to achieve in a limited time frame.

The first two questions evaluated the caregiver’s impression of the patient’s overall function during the past year, and their expectations for the next two years (see appendix for details of the questionnaire).

The second set of questions included open-ended inquiries that were, according to the caregivers, the most important activities/functions of daily living that they hoped would be maintained or gained in their children.

Caregivers were finally asked to provide information on their expectations regarding clinical trials.

They were informed on the next three items that their child could not achieve on the HFMSE scale, asking more specifically, if achieving at least one of these abilities would justify their participation in a clinical trial.

The last question enquired whether the caregivers would consider having their child take part in a potential trial in the presence of mild side-effects. A trained clinician conducted the in-person interviews and telephone interviews using a semi-structured data collection sheet. The interviews lasted 15-20 min on average. The questions covered caregivers’ views and expectations regarding a possible participation in a clinical trial.

*Statistical Analysis*: Responses of the non-ambulant and ambulant groups were compared for significant difference using the Wilcoxon-Mann-Whitney test. A *p* value of <0.05 was considered significant.

For the question assessing whether parents would consider entering in a study if their child could achive at least 1 (score0), two (score 1) or more than 2 activities on the HFMSE, a Chi-square analysis was used to was used to correlate the level of responses (0, 1, 2) with functional scores. A *p* value of <0.05 was considered significant.

## Results

### Part 1: Content validity

Sixty-three individuals participated in the focus groups. These included 30 caregivers and 25 patients. Eight professionals (psychologists, Physical Therapists or clinicians) conducted the interviews and facilitated the discussion by introducing the items without contributing to data collection in an effort to avoid bias.

Patient ages ranged from 14 to 35 years, 3 were ambulant and 22 non ambulant (20 type 2 and 2 type 3).

The caregivers were all parents (17 mothers and 13 fathers). The age of the patients represented by the caregivers ranged between 2 and 26 years, 5 were ambulant and 25 non ambulant (all type 2). Only one parent/caregiver was allowed to participate for each patient.

The caregivers commented on all the functional scale items and provided a comparison to activities of daily living for each of them. Table 1 shows the responses in the 4 focus groups illustrating whether some responses were reported in more than one focus group. Many activities (64.07%) were suggested by more than one group with only 37 of the 103 activities suggested by one group only. Of these 37, only 7 were suggested by the group including patients.

**Table 1:** Details of the caregivers and patients’ responses in the 4 focus groups

HMFSE Item	HMFSE activities	Answers	Group 1	Group 2	Group 3	Group 4
1	Able to sit on chair or with legs off bed with or without hand support	Sitting on normal school chair or public spaces (stools in restaurant)	●	●	●	●
		Sitting on toilet	●	●		●
		Sitting in car			●	
		Independence out of the house	●			●
		Dress by herself/himself		●		
2	Able to sit on floor cross legged or legs stretched in front	Play on floor with siblings	●	●	●	●
		Sit on lounge chair, deck-chair		●		●
		Picnic			●	●
		Travel with less equipment	●			
		Inclusion in activities		●		
3	Able to bring hands to face at eye level	Wash face	●	●	●	●
		Brush and style	●	●	●	●
		Eat	●		●	
		Put on eye glasses	●	●		●
		Answer telephone			●	
		Blow nose	●			
4	Able to bring hands to head	Scratch head	●	●	●	●
		Wash, brush, style hair		●	●	●
		Put on hat	●	●		●
		Dress upper body	●		●	
5	Roll to side	Sleep by myself in my own room		●	●	
		Caregiver does not have to wake up to turn him/her	●	●		●
		Help during dressing lying down		●	●	
		Not having to turn head to see	●			
6-7-8-9	Roll	Play	●	●		
		Sleep well		●	●	
		Sunbathe		●	●	
		Experience space	●			●
		Reach for something at sides when lying down		●		●
10	Able to lye down from sitting	Independence: lye down and rest when tired	●	●	●	●
		Fun movement when falling	●	●		
		Rest on the back			●	
		Safety: Fall in a controlled way (avoid head trauma)	●			
11	Able to raise head when lying prone	Turn head react to stimulus, visual exploration of surroundings	●	●	●	
		Read a book	●		●	●
		Not be afraid of choking		●		
		Watch TV		●		●
		On beach not get sand in face	●			
12-13	Able to prop on forearms or extend arms	Read a book	●	●	●	
		Watch TV		●	●	●
		Stretch back	●			
		Sun bathe			●	
14	Able to sit up from lying	No need for assistant	●	●	●	
		Wake up and not have to wait for someone to sit me up	●	●	●	●
		Independence	●	●		
		Sit up and drink at night	●			●
15	Able to four-point kneel	Play like an animal in school	●		●	●
		Hiding				●
		Be able to fit under small spaces				●
16	Able to crawl	Move around	●	●	●	●
		Experience space		●	●	
		Go get objects	●	●		
		Play on floor			●	●
17	Lift head from supine	Change head position	●	●	●	
		Drink at night			●	●
		Read	●			
		Watch TV		●		
		Check the clock or alarm				●
18	Stand with support	Use toilet standing (boy)	●		●	●
		Use full length mirror, perceive body dimensions and proportions	●	●		
		Shower properly	●			
		Climb in car			●	
		Use kitchen burners, cook		●		
19	Stand without support	Public spaces: wait for bus, stand in cue		●		
		Cook			●	
		Use normal sink	●			
		Dress			●	
		Reach something on a shelf				●
20	Able to walk	Freedom	●	●	●	
		Go where and when you please	●	●	●	
		Get to places	●	●	●	
		Not to have to rely on wheelchair batteries			●	
21-22	Able to flex hip from supine	Dress (pants, socks)	●	●	●	●
		Scratch legs, kill mosquito	●	●		
		Change position			●	
23-24-25-26	Able to half kneel	Pick up object on floor		●	●	
		Tie shoe laces	●	●		
		Put away object on low surfaces	●			
		Pet a dog		●		
		Play			●	
		Make a proposal				●
		Kneel in church				●
		Talk with a kid				●
27	Able to go from standing to sitting	Not get hurt when falling or not fall in an embarrassing way	●		●	
		Sit on grass or sand	●	●		●
		Pet a dog			●	
		Sit beside a friend in same position/play on floor	●		●	
		Pick up something from floor		●		●
28	Able to squat	Sit when needed	●		●	
		Pick up objects on floor		●	●	●
		Pee	●			●
		Tie shoes			●	
		Pull up trousers	●			
29	Able to jump	Have fun, play	●	●	●	●
		Dance, gymnastics		●	●	
		Avoid obstacles	●	●		●
		Normality	●		●	
		Go to friends’ home regardless of where they live	●		●	●
		Stay and live in my own home		●		
30-31-32-33	Go up and down stairs	Absence of barriers	●	●	●	●
		Normality	●	●		
		Go to friends’ home regardless of where they live	●	●		●
		Stay and live in my own home			●	

### Part 2

One hundred-forty-nine of the 151 caregivers who were invited to participate agreed to complete the questionnaire (response rate 98.7%). The caregivers were all parents (Additional file 1).

The patient ages ranged from 17 months to 30 years. Thirty-three patients were ambulant and 116 non ambulant (109 type 2 and 7 type 3).

When asked to describe the patients’ clinical course over the last year, 15% reported stability, 72% deterioration and 12% improvement.

**Fig. 1:**
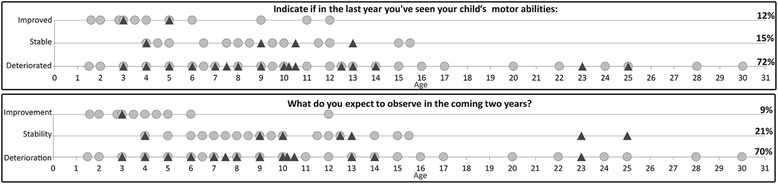
Individual responses plotted against age in non-ambulant (gray circle) and ambulant (▲) patients

When asked what to expect in the next 2 years, 21% anticipated a stable course, 70% a deterioration and 9% an improvement. Figure 1 summarizes the distribution of findings for both questions.

When asked to summarize their expectations regarding clinical trials, 72% of the caregivers would participate if the treatment slowed down deterioration, 88% if it would stop deterioration and 97% if the treatment produced an improvement.

**Fig. 2:**
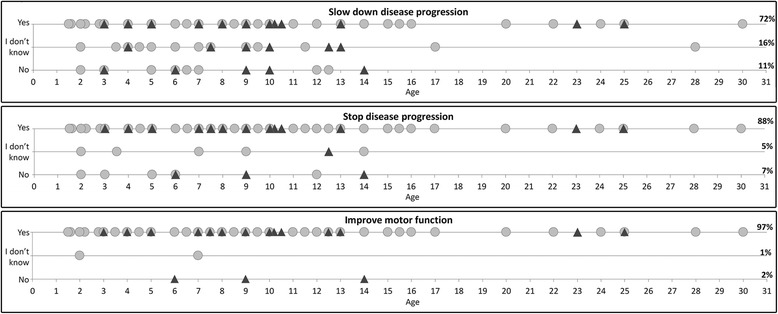
Individual responses to the question: ‘Would you agree to have your child take part in a potential trial if, in the absence of side-effects or with possible minimal side-effects, the prospect was to slow down a possible decline in motor function for at least two years?’

**Fig. 3:**
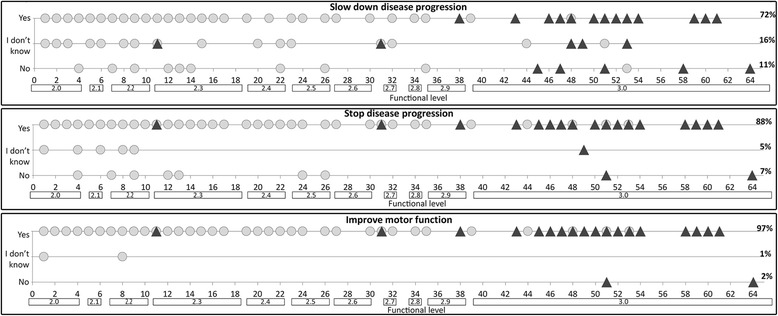
Individual responses to the same question as in fig. 2. Responses are plotted against functional level for non ambulant (gray circle) and ambulant (▲) patients. Functional level is defined both using the raw HFMSE scores and the classification expressing severity in decimals, starting from 2.1, for patients who are just able to sit, to the strongest type 2, 2, who are able to stand but not to walk, to the type 3 [1]

When we correlated the responses to the functional status of the patients, the percentage of caregivers willing to take part in a clinical trial, if the treatment was expected to slow down deterioration, was higher in the non-ambulant group (76%) than in the ambulant group (61%) even though the difference was not significant (*p* > 0.05) (Figure 2 and 3).

When asked, after being informed of the next three items that their child could not achieve on the HFMSE scale, if achieving at least one of these abilities completely (score 2) would justify their participation in a clinical trial, 75% would consider taking part if they could achieve at least one of these abilities, 89% if they could achieve 2 and 100% if they could achieve more than 2. The results were widely distributed across functional levels and age. The correlation between the responses and the functional scores was not significant (*p* > 0.05).

**Fig. 4:**
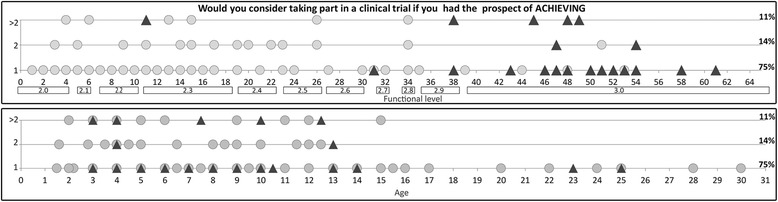
Individual responses plotted against age and functional level in non-ambulant (gray cirlce) and ambulant (▲) patients

The percentage of caregivers considered participating in a clinical trial if their child might achieve one activity was not significantly different among the ambulant and non ambulant groups (*p* > 0.005), a list of of the most frequent activities that caregivers hope will be achieved is provided in Table 2. The results were widely distributed across functional levels and age (Fig. 4).

**Table 2:** Details of the most frequent activities that caregivers hope will be achieved

Activities to achieve	%	Activities to achieve	%
Strength in the upper limbs	15,7%	Stand up from a chair	2,5%
Rolling	9,0%	Stand up from floor	2,5%
Walking	7,1%	Respiratory function	2,2%
Standing independently	6,2%	Writing skills	2,2%
Strength of the head	5,6%	Run	1,9%
Personal hygiene	4,9%	General autonomy	1,5%
Move independently	4,9%	Crawling	1,5%
Do stairs	4,6%	Hop/Jump	1,5%
Eat independently	3,1%	Strength of the hands	1,5%
Sit independently	3,1%	Use manual wheel-chair	1,2%
Strength in the lower limbs	2,8%	Balance	1,2%
Strength of the trunk	2,8%	Standing with support	1,2%

## Discussion

The results of our first study, assessing content validity, confirm that the activities of the HFMSE, known to be relevant both in clinical and research practice, are also clinically meaningful to patients and their caregivers. Following the FDA guidelines [18], we used a questionnaire exploring all items and a structured qualitative interview as part of focus groups, in a cohort of patients and caregivers that included both genders, patients of different ages, and SMA patients who represent the full range of motor function captured on the HFMSE. The analysis of the transcripts of the focus groups demonstrated that each activity included in the HFMSE was related to activities of daily living that were relevant to patients and their caregivers, as often suggested by many participants in more than one focus group. The group including patients had similar responses to the other 3 groups, only including parents, for 101 of the 103 responses provided. Each of the items and the explored domains were thought to be appropriate for use in SMA.

Not surprisingly, as also demonstrated in the second part of our study, the responses of patients and caregivers showed a degree of heterogeneity. This can be easily explained by the fact that the patients included in our study had a wide age range, from infants below age 2 years to adults in their thirties, and variable functional levels, from very weak patients with a HFMSE score of 0 who could only sit very briefly to strong ambulant patients who achieved the highest scores on the scale. It is, therefore, expected that the responses of individual patients/caregivers focused on the activities that were most challenging for them/their child, according to their respective functional levels.

In the second part of our study we also explored the perception of the families regarding their child’s disease course with respect to motor function. The majority (72%) felt that over the last year their child had deterioration, whereas 15% reported a stabilization. The remaining 12% reported an improvement, and this occurred mainly in the younger end of the cohort. These results are in agreement with our recent collaborative study showing that a clinical improvement could be detected mainly in young children up to age 6 years as documented on the HFMSE. Clinical deterioration was more likely to occur around puberty [10].

As a result, over 70% of the caregivers felt that they would consider participating in a clinical trial if, in the absence of significant side effects, the intervention would slow down the rate of deterioration. Not surprisingly there were even higher percentages of caregivers considering participating in a trial if the prospect was stabilization (88%) or improvement (97%). These results should be interpreted with caution as considering participation in a clinical trial is complex and not all the studies have the same demands or the same possible outcomes. Nevertheless, these findings are already, in and of themselves, strongly indicative that caregivers would consider a trial even if the prospect was limited to influencing the rate of deterioration regardless of age or functional level.

In the second part of this study we tried to explore, in further detail, whether achieving or maintaining activities on the HFMSE had the same relevance regardless of HFMSE scores and, therefore, different functional levels.

The advantage of this approach is that the caregivers could relate the questions to the actual status of the child and were asked questions about activities that were realistically close to their child’s possible achievements rather than generic questions on activities, such as walking or running that would be highly desirable but, at least in a limited time frame, difficult or impossible to achieve especially for the weakest patients. When asked if they would consider taking part in a trial if there was the possibility of achieving one, two, or more than two activities, 75% considered participation even if just one activity was achieved. These results were widely distributed across functional levels and age.

## Conclusions

These findings suggest that even if the achievable activities are different, any improvement is considered to be meaningful, regardless as to whether the baseline score is very low, in the middle, or very high. This conclusion is particularly important considering the fact that the ordinal nature of the scale makes this comparison difficult.

This study has several limitations; first, the number of patients was relatively small but the range of age and functional level was quite wide and representative of the ambulant and non-ambulant SMA population. Since the Italian and English versions of the questionnaire’s data collection sheet, and the forms used to illustrate the items with pictures from the scale manual, were piloted and validated before their use, these findings can justify another follow-on study with a larger cohort.

All the patients were followed in tertiary care centers or were part of advocacy groups and were unlikely to be representative of a more general population. While this is a potential bias, these patients are more likely to be representative of a trial population with appropriate standards of care and level of information and participation. Another apparent limitation is the fact that, in the second part of the study, we only involved caregivers. This was, however, necessary in order to include patients of all ages; very young patients would have not been able to complete the questionnaires. We acknowledge that the patient’s perspective is however very important and further studies are in progress to collect data directly from patients who are older than age 12 years.

Despite these limitations, the study results support the use of the HFMSE as a robust outcome measure in clinical trials, not only because all the individual items appear to be meaningful to patients and caregivers, but also because even small changes detected on the scale appear to be relevant and to justify participation in a clinical trial. The great majority of the caregivers would already consider participation of their children in a clinical study even if the best outcome would be just to reduce deterioration. Of course, even more caregivers would agree to their child’s participation if the prospect was to remain stable or improve; however, it is of interest that even when aiming for an improvement, a small improvement (just one activity) would already be sufficient in their mind to justify participation in a trial with an investigational drug.

Finally, these results are important as they provide the views of patients and caregivers and complement other studies currently being performed and designed to establish item response theory approach and the minimally important difference using statistical analysis.

## Additional file

Additional file 1: Questionnaire provided to the carers. Sample of the questionnaire submitted to the carers. (DOCX 19 kb)

## Abbreviations

FDA: Food and Drug Administration.
HFMSE: Hammersmith Functional Motor Scale Expanded
SMA: Spinal muscular atrophy
