# Supplementary material for: Content validity and clinical meaningfulness of the HFMSE in spinal muscular atrophy
Source: BMC Neurol. 2017 Feb 23;17:39. doi: 10.1186/s12883-017-0790-9 (PMC5324197; doi:10.1186/s12883-017-0790-9)
Supplement: Additional file 1: — Questionnaire provided to the carers. Sample of the questionnaire submitted to the carers. (DOCX 19 kb) [file 12883_2017_790_MOESM1_ESM.docx]

**Additional File 1**

*Questionnaire provided to the carers*

*Please indicate if in the last year your child*

Has remained stable 🌕

Has experienced deterioration 🌕

Has improved some abilities 🌕

*What do you expect in the coming two years?*

To remain stable 🌕

To experience deterioration 🌕

To improve some abilities 🌕

*In your view, what are the most important activities/functions of daily living that you would like your child to maintain in order to keep his/her standards of everyday living? Can you list at least 3 of them?*

*In your view, what are the most important activities/functions of daily living that you would like your child to gain in order to improve his/her everyday life? Can you list at least 3 of them?*

*Would you agree to have your child take part in a potential trial if, in the absence of side effects or with possible minimal side effects, the prospective was to slow down a possible decline in motor function for at least two years?*

To slow down a possible decline in motor function? Yes 🌕 No 🌕 I don’t know 🌕

To stop a possible decline in motor function? Yes 🌕 No 🌕 I don’t know 🌕

To improve motor function? Yes 🌕 No 🌕 I don’t know 🌕

*Referring to the scale performed, there were some activities that could not be achieved **

1) _____________________________________________________________

2) _____________________________________________________________

3) _____________________________________________________________

** the first 3 items of the HFMSE scored as 0 are recorded by the physical therapist before the questionnaire is given to the carers*

*Do you think that achieving one or more of these activities would represent a meaningful change in his/her daily life*

*Would you consider participating in a clinical trial if your child had the prospective of achieving:*

At least one of these abilities^*^

At least two abilities*

More than two abilities*

*referring to the three abilities listed above

*Would you agree to have your child take part in a potential trial if, in the absence of side effects or with possible minimal side effects, the prospective was to stop a possible decline in motor function*

Yes 🌕 No 🌕 I don’t know 🌕
